# Secondary Metabolites from Fungi—In Honor of Prof. Dr. Ji-Kai Liu’s 60th Birthday

**DOI:** 10.3390/jof8121271

**Published:** 2022-12-01

**Authors:** Tao Feng, Frank Surup

**Affiliations:** 1 School of Pharmaceutical Sciences, South-Central Minzu University, Wuhan 430074, China; 2 Department of Microbial Drugs, Helmholtz Centre for Infection Research GmbH, 38124 Braunschweig, Germany

It is our pleasure and privilege to serve as Guest Editors for this Special Issue of the *Journal of Fungi* in honor of Professor Ji-Kai Liu’s 60th birthday. We want to take this opportunity to commemorate his outstanding contribution to the field of fungal natural products chemistry, especially higher fungal chemistry. This Special Issue includes contributions from friends, collaborators, and many colleagues from the field of natural product chemistry.

Liu was born in Wuwei, Anhui province, People’s Republic of China. He graduated with a Ph.D. from Lanzhou University in 1988, and in the same year, started an independent position at Sun Yat-Sen University (Zhongshan University), first as a lecturer, then an associate professor in 1992, rising through the ranks to full professorship in 1995. After three years in Germany working with his friend Marc Stadler (Saarland University and Bayer Pharma Research Center), Liu moved to Kunming Institute of Botany, Chinese Academy of Sciences, in 1997, where he started his career in higher fungal chemistry.

With more than 30 years of achievements, Liu’s work touches all areas of fungal natural product research. He has pioneered several directions that are being studied by scientists around the world, including the beginning of important classes of compounds such as mycotoxins, fungal pigments, and fungal nitrogenous compounds [1,2,3,4]. Liu’s team has investigated the chemical constituents of at least 300 fungal species worldwide. More than 4000 fungal natural products, including 2000 novel ones, have been established from basidiomycetes and ascomycetes.

Liu focuses on fungi toxins. Over the past 30 years, Yunnan sudden death syndrome has been responsible for more than 300 deaths in Yunnan Province, southwest China. It has long been a mystery and a fascinating problem. Liu’s team identified two unusual amino acids as new toxins from the mushroom *T. venenata*. Mice treated with both amino acids exhibited increased serum creatine kinase (CK) activity. These new toxins are the cause of Yunnan sudden death syndrome, an important local epidemic of undefined etiology [5,6,7]. A campaign to warn people against eating this tiny mushroom with printed brochures has dramatically reduced the number of deaths, with no deaths reported from 2010 to 2014. This research has saved the lives of more than 80 people. In the south of France, twelve people were hospitalized for severe weakness and muscle loss after eating wild mushrooms [8]. Liu’s team identified the toxins found in a previously unknown poisonous European mushroom *Tricholoma terreum*. Fifteen novel triterpenoids were isolated from the fruiting bodies of *T. terreum*. Two abundant compounds in the mushroom displayed acute toxicity when administered orally in mice, and both of them were found to increase serum creatine kinase levels in mice, indicating that *T. terreum* may be the cause of mushroom poisoning, ultimately leading to rhabdomyolysis [9]. 

Liu’s team discovered vibralactone, an unusual fused β-lactone-type metabolite, from the basidiomycete *Boreostereum vibrans* in 2006. This molecule exhibited good inhibitory activity against pancreatic lipase [10]. The structure was optimized using vibralactone as the original template and more than 200 derivatives have been synthesized. Moreover, a new derivative with significant pancreatic lipase inhibitory activity (IC_50_ = 14 nM) has been considered to be a drug candidate. A unique biosynthetic pathway of vibralactone, including several very interesting reactions that may involve unusual enzymes, was elucidated [11,12,13]. 

The fungal natural products with structural and biological activity diversity discovered by Liu’s team are too numerous to list. More than twenty molecules have been rated “hot off the press”, playing important roles in synthetic chemistry, pharmacology, and drug development [14,15,16,17,18,19,20,21,22,23,24,25,26,27,28,29,30,31,32,33,34,35,36,37,38].

Liu’s workload and achievements are so broad that describing them in a brief editorial is hard. It can be said that Liu has left an indelible mark on the field, with over 450 publications and over 100 trainees from academia and industry who have carried on Liu’s passion for fungal natural products. We hope that the above description provides at least an overview of Liu’s main discoveries and his enormous impact on the field of higher fungal natural products. Nonetheless, at 60, Liu is still devoted to the work of fungal natural products chemistry with full enthusiasm. His professionalism and positive attitude toward life have deeply affected his students, colleagues, and fellow researchers. Therefore, it is with the deepest respect and affection for Liu that we, on Liu’s 60th birthday, publish this Special Issue of the *Journal of Fungi* in Liu’s name. We look forward to the exciting new discoveries reported in the future by Liu’s team and the labs of Liu’s scientific family.

Liu’s comprehensive research interests are reflected by the broad scope of the contributions in this SI, covered by six reviews and sixteen original research articles.

Terpenoids are among the most important class of natural products and are thus the subject of two review articles. Whereas Dai et al. review the isolation, structural determination, bioactivities, and synthesis of sesquiterpenoids produced by fungi over the past five years [39], Fa-Lei Zhang et al. provide an overview of the structures, biological activities, evolution, organic synthesis, and biosynthesis of fungal diterpenoids reported in the period from 2010 to 2020 [40]. 

The review of Wen et al. underlines the importance of endophytic fungi as an alternative source of secondary metabolites, some of which have potential in the development of new pharmacologicals [41]. 

Nearer in its scope are the sulfur-containing compounds from plant endophytic fungi. Fan et al. reported 143 new sulfur-containing compounds that were reported from 1985 to 2022 and their fungal producers, plant sources, chemical structures, and bioactivities. These natural products mainly belong to the classes of polyketides, nonribosomal peptides, terpenoids, and hybrids [42]. 

The review of Hou et al. covers sorbicillinoids, a family of hexaketide metabolites with a characteristic sorbyl side-chain residue. Sixty-nine sorbicillinoids from fungi, newly identified from 2016 to 2021, are summarized in this review, including their structures and bioactivities [43].

Last but not least, Dai et al. report on the biosynthesis of fungal natural products, which involves two separate pathway crosstalk. This stresses that fungal natural product biosynthetic genes are not always arranged simply within one single biosynthetic gene cluster, thus increasing the structural complexity and chemical diversity of fungal NPs and expanding the scope of bioactivities [44].

For the original research articles, new natural products from Ascomycota are the first focus of this SI. Both Garcia et al. and Yan et al. report new cytochalasans from *Sparticola triseptata* and the endophytic fungus *Phomopsis* sp. [45,46]. These showed antiproliferative, cytotoxic, and anti-migratory activities, respectively, as expected for these PKS-NRPS hybrids. 

During screening for new natural products with inhibitory activities on acetylcholinesterase, the endophytic fungus *Phaeosphaeria* sp., isolated from *Huperzia serrata,* was investigated, and new small polyketides were discovered by Xiao et al. [47]. 

Verrucosidin is a toxic pyrone-type mycotoxin of polyketide origin. New derivatives were discovered by Han et al. from *Penicillium cellarum* using a state-of-the-art MS/MS-based molecular networking approach [48]. 

New antibacterial chloro-containing polyketides from the alga-derived fungus *Asteromyces cruciatus* were elucidated by Zhuravleva et al. The new isoprenylated cyclohexanol structures contain a characteristic conjugated alkyne–alkene moiety, presumably responsible for the observed activities [49]. 

From another marine-derived fungus, *Acremonium chrysogenum*, Duan et al. obtained sorbicillinoid derivatives with radical scavenging activities. The analysis of the biosynthetic gene cluster, the proposed biosynthetic pathway, and the radical scavenging activity complete the study [50].

Further studies on endophytic fungi by Ai et al. and Yu et al. report new eremophilane sesquiterpenoids and new sativene, as well as longifolene sesquiterpenoids plus two new xanthones from *Boeremia exigua* isolated from *Fritillaria hupehensis* and the Kiwifruit-associated fungus *Bipolaris* sp., highlighting the vast chemical diversity to be explored by these plant-associated fungi [51,52].

Three manuscripts report on the isolation of secondary metabolites from Basidiomycota. First, Dai et al. reported eighteen previously undescribed bergamotane sesquiterpenes and one new victoxinine derivative from the edible mushroom *Craterellus odoratus*. Some of the compounds possess immunosuppressive activity [53].

Peng et al. reported on anti-adipogenic lanostane-type triterpenoids from the edible mushroom *Ganoderma applanatum*, which is utilized for medical purpose [54]. Other lanostane-type metabolites are presented from the species *Ganoderma australe* by Lin Zhou [55]. 

The phenalenone skeleton, found in several plant or fungal compounds, presumably plays a role in protecting these organisms against various external threats. Ibrahim et al. explored the activity of fungal phenalenone derivatives as potential CK2 inhibitors using computational methods [56].

The genetic engineering of fungal natural product biosynthesis genes and gene clusters constitutes the last focus of our SI. Feng et al. engineered *Aspergillus oryzae* for the heterologous expression of bacterial polyketide synthase genes, having a modular architecture [57]. 

The biosynthetic gene cluster encoding ilicicolin biosynthesis from the well-studied organism *Trichoderma reesei* was studied by heterologous expression in the fungal host *Aspergillus oryzae* by Shenouda et al. from the Cox group [58]. Furthermore, they developed *Trichoderma reesei* as a microbial cell factory for the heterologous expression of secondary metabolites for waste valorization [59].

After revealing the biosynthetic pathway of the potential protein kinase C inhibitor balanol through overexpression in previous studies, Li et al. now report the regulation of balanol biosynthesis by BlnR. This further improved the titers of balanol in the herb fungus *Tolypocladium ophioglossoides* [60].

Finally, we thank all of the contributors to this Special Issue and warmheartedly wish Prof. Ji-Kai Liu all the best for the future.
